# Predictive levels of vascular endothelial growth factor (VEGF), thymidine kinase 1 (TK1) with interleukin-6 (IL-6), plasma T cells, NK cells as well as B cells in treating diffuse large B-cell lymphoma receiving rituximab

**DOI:** 10.5937/jomb0-54911

**Published:** 2025-07-04

**Authors:** Lihua Tian, Baoan Luo, Jun Tang, Jiagui Ye

**Affiliations:** 1 Lujiang County People's Hospital, Department of Hematology, Anhui Hefei, China; 2 Lujiang County People's Hospital, Department of Oncology, Anhui Hefei, China

**Keywords:** diffuse large B-cell lymphoma, rituximab, chemotherapy, lymphocyte subsets index, difuzni veliki B-ćelijski limfom, rituksimab, hemoterapija, indeks podgrupa limfocita

## Abstract

**Background:**

The aim was to explore the effect of rituximab in combination with chemotherapy in treating diffuse large B-cell lymphoma and levels of vascular endothelial growth factor (VEGF), thymidine kinase 1(TK1) with interleukin-6 (IL-6), plasma T cells, NK cells as well as B cells.

**Methods:**

Eighty patients admitted to Lujiang County People's Hospital from January 2022 to January 2024 were included. The control group accepted cyclophosphamide, doxorubicin, vincristine, and prednisone chemotherapy regimens. The research group was treated with rituximab based on the control group. The clinical effects, vascular endothelial growth factor (VEGF), thymidine kinase 1 (TK1) and interleukin-6 (IL-6) levels, lymphocyte subsets index, quality of life and occurrence of adverse reactions were compared in both groups.

**Results:**

The research group's total clinical effective rate was better than the control group's (P<0.05). After therapy, compared to the control group, vascular endothelial growth factor, thymidine kinase 1, and interleukin-6 levels in the research group presented lower (P<0.05), plasma T cells, natural killer cells along with B cells in the research group presented lower (P<0.05), and Quality of Life Core Questionnaire-Core 30 scores in the research group presented higher (P<0.05). There was no difference in adverse reactions between the two groups (P>0.05).

**Conclusions:**

Rituximab combined with chemotherapy is effective in treating DLBCL patients, which can reduce serum-related factors promoting immune function and quality of life. Our study may provide compelling evidence for supporting the therapeutic regimen of rituximab combined with chemotherapy in DLBCL patients.

## Abstract (sr)

**Uvod:**

Cilj je bio da se ispita efekat rituksimaba u kombinaciji sa hemoterapijom u lečenju difuznog velikog B-ćelijskog limfoma, kao i nivoa vaskularnog endotelnog faktora rasta (VEGF), timidinske kinaze 1 (TK1) sa inter leukinom-6 (IL-6), plazma T ćelijama, NK ćelijama i B ćelijama.

**Metode:**

U istraživanje je bilo uključeno 80 pacijenata primljenih u Narodnu bolnicu okruga Lujiang od januara 2022. do januara 2024. Kontrolna grupa je primala hemoterapijski režim koji je uključivao ciklofosfamid, dokso-rubicin, vinkristin i prednizon. Grupa ispitanika je, pored terapije kontrolne grupe, bila lečena i rituksimabom. Upoređivani su klinički efekti, nivoi vaskularnog endotelnog faktora rasta (VEGF), timidinske kinaze 1 (TK1) i interleukina-6 (IL-6), indeks podgrupa limfocita, kvalitet života i učestalost neželjenih reakcija kod obe grupe.

**Rezultati:**

Ukupna klinička efikasnost u grupi ispitanika je bila bolja nego u kontrolnoj grupi (P<0,05). Nakon terapije, u poređenju sa kontrolnom grupom, nivoi vaskularnog endotelnog faktora rasta, timidinske kinaze 1 i interleukina-6 u grupi ispitanika su bili niži (P<0,05). Nivoi plazma T ćelija, prirodnih ćelija ubica (NK ćelija) i B ćelija su takođe bili niži u grupi ispitanika (P<0,05). Rezultati upitnika o kvalitetu života (Core 30) su u grupi ispitanika bili viši (P<0,05). Nije bilo razlike u učestalosti neželjenih reakcija između dve grupe (P>0,05).

**Zaključak:**

Rituksimab u kombinaciji sa hemoterapijom je efika san u lečenju pacijenata sa DLBCL, jer može da smanji se rumske faktore povezane sa imunitetom, da poboljša funkciju imunog sistema i kvalitet života. Naša studija može da pruži ubedljive dokaze koji govore u prilog terapijskog režima rituksimaba u kombinaciji sa hemoterapijom kod pacijenata sa DLBCL.

## Introduction

Diffuse large B-cell lymphoma (DLBCL) is the most common pathological type of non-Hodgkin’s lymphoma (NHL), accounting for almost one-third of all NHL cases [1]. DLBCL is a highly aggressive tumour primarily from B lymphocytes, which play a key role in the immune system [2]. DLBCL can spread to all body parts, including lymph nodes, bone marrow, spleen, and liver, seriously threatening patients’ quality of life and health [3]. The incidence of DLBCL in the world is very high. According to relevant statistics, the incidence of DLBCL in Asia can be as high as 40% [4]. Because the pathogenic factors of DLBCL are still not clearly understood, DLBCL has been on the rise in recent years [5].

At present, there are many chemotherapy regimens for DLBCL, among which the CHOP regimen (cyclophosphamide, doxorubicin, vincristine, along with prednisone) is considered to be the standard chemo therapy regimen for DLBCL [6]. The overall response rate of this regimen is 80%–90%, of which the complete response (CR) rate is 40%–50%, and the five-year overall survival rate is about 30% to 40% [7] [8]. Most patients are insensitive to it, and it is challenging to achieve CR or relapse after remission; follow-up treatment is complex, especially in IPI high/medium high-risk patients, and the long-term survival rate is only about 20% [9]. Therefore, it is urgent to investigate new treatment options to improve cure rates and prolong survival in DLBCL patients.

With the in-depth development of molecular biology, immunology, and genetic engineering, most B-cell lymphoma cells have been found to express CD20 antigen positively, and targeted therapy for CD20 antigen has become a research hotspot in this field [10]. Rituximab belongs to a human/mouse chimeric monoclonal antibody against B-cell CD20 antigen developed by genetic engineering, which can specifically bind to B-cell CD20 antigen [11]. After binding to the antigen, rituximab kills lymphoma cells by inducing antibody-dependent cytotoxicity, inducing complement-dependent cytotoxicity, and directly inhibiting cell growth or inducing cell apoptosis [12]. Rituximab combined with chemotherapy is currently the first choice for the treatment of DLBCL [13] [14].

Therefore, our study was intended to explore the impact of rituximab in combination with chemotherapy in treating DLBCL. Our study shows that rituximab combined with chemotherapy is effective in DLBCL patients. This may provide strong evidence to support a treatment regimen for rituximab combined with chemotherapy in DLBCL patients.

## Materials and methods

### General data

Eighty DLBCL patients admitted to our hospital from January 2022 to January 2024 were chosen as study objects. Inclusion criteria: (1) All patients met the diagnostic criteria related to DLBCL in the 2007 First Edition of Treatment Guidelines for Non-Hodgkin Lymphoma published by the National Comprehensive Cancer Network of the United States; (2) Patients were confirmed as CD20-positive by routine histopathological examination. (3) Expected survival was at least 3 months; (4) Patients signed informed consent. Exclusion criteria: (1) Patients with other types of NHL; (2) Allergic to investigational drugs; (3) Patients who had experienced autologous peripheral blood stem cell transplantation; (4) Complicated with serious organic diseases (heart, lung, liver, kidney); (5) Combined with other malignant tumours; (6) Pregnant or lactating women. Patients were assigned to the control group (CG) and research group (RG) following the treatment plan, with 40 patients in each group.

### Methods

The CG was treated with the CHOP chemotherapy regimen as follows: cyclophosphamide (cyclophosphamide for injection, Jiangsu Shengdi Pharmaceutical Co., LTD., specification: 0.2 g × 200) was administered through intravenous infusion on the first day with a dose of 750 mg/m^2^. Doxorubicin hydrochloride for injection (Doxorubicin hydrochloride for injection, Shanxi Pude Pharmaceutical Co., LTD., specification: 10 mg/10 doses/box) was admi nistered intravenously on the first day with a dose of 50 mg/m^2^. Vincristine (Vincristine sulfate for injection, Shaanxi Boson Biopharmaceutical Group Co., LTD., specification: 1 mg) was administered intravenically on the first day with a 1.4 mg/m^2^ dose. Prednisone was orally administered 100 mg daily for 5 days.

Based on the CG, the RG was treated with rituximab. Intramuscular ranitidine injection (Hangzhou Minsheng Pharmaceutical Co., LTD., specification: 2 ml: 50 mg) was performed 30 minutes before administration to prevent allergic reactions. Then the patient was intramuscularly injected with promethazine hydrochloride injection (Shanghai Hefeng Pharmaceutical Co., LTD. (domestic), specification: 25 mg/1 mL) 25 mg and dexamethasone (Dexamethasone acetate tablet, Tianjin Pacific Pharmaceutical Co., LTD., specification: 0.75 mg/tablet) 20 mg oral administration. Next, the patient was given rituximab (Rituximab injection, F. Hoffmann-La Roche Ltd., Specification: 100 mg/10 mL × 1 bottle/box), the dosage was 375 mg/m^2^. The other treatments were the same as the CG.

Four cycles of chemotherapy were performed at 21 days per cycle.

### Observation indicators

(1) The evaluation indexes of efficacy were separated into four grades: complete response (CR), partial response (PR), stable disease (SD) as well as progressive disease (PD), following the WHO evaluation criteria of the efficacy of solid tumours. The specific criteria were: CR: the tumour disappeared after clinical diagnosis; PR: The product of maximum tumour diameter and maximum vertical diameter was lessened by 50%; SR: The product of maximum tumour diameter and maximum vertical diameter was reduced by <50%; PD: The product of the maximum diameter of the tumour and its maximum vertical diameter >25% or a new lesion appeared. Total effective rate = CR%+PR%.

(2) Serum related factors. 4 mL of fasting venous blood was obtained from patients. After centrifugation, the serum was separated. The levels of vascular endothelial growth factor (VEGF), thymidine kinase 1 (TK1) and interleukin-6 (IL-6) were detected by ELISA kit (Shenzhen Jingmei Biotechnology Co., LTD., Shenzhen, China).

(3) Lymphocyte subsets index. Blood samples of the two groups were collected, and the two groups’ plasma T cells, NK cells, and B cells were detected by flow cytometry (Thermo Fisher, USA).

(4) The occurrence of adverse reactions (thrombocytopenia, neutropenia, mild anaemia, hepatotoxicity, nausea and vomiting, and neurotoxicity) was recorded in the two groups.

(5) Quality of life. Quality of Life QuestionnaireCore 30 (QLQ-C30) [14] could be adopted to evaluate the quality of life of patients, which includes 5 areas: physical function, emotional function, cognitive function, role function, as well as social function. The higher score represented a better quality of life.

### Statistical analysis

Statistical software SPSS 17.0 was implemented to process the data. The data were exhibited as (n, %), and the measurement data were exhibited as (mean ± standard deviation). The groups were compared by χ^2^ test and t-test, respectively. P<0.05 meant the difference was statistically significant.

## Results

### Demographic data in both groups

No significant difference was discovered in gender, age, tumour stage, radiotherapy history and bone marrow invasion between the two groups (P>0.05, Table 1), which showed comparability.

**Table 1:** General data of patients in both groups

Index		Control group (n=40)	Research group (n=40)	P
Gender (male/female)		18/22	17/23	>0.05
Age (years)		54.27±9.65	54.32±10.23	>0.05
Tumor stage	Stage I-II	19	18	>0.05
	Stage III-IV	21	22	
Radiotherapy history	Yes	5	6	>0.05
	No	35	34	
Bone<br>marrow invasion	Yes	3	4	>0.05
	No	37	36	

### Clinical effect of two groups

Table 2 displayed that the total clinical effective rate of the RG was 80.00%, and that of the CG was 55.00%. The total clinical effective rate of the RG was higher than that of the CG (P<0.05).

**Table 2:** Clinical effect of two groups.

Groups	Cases	CR	PR	SD	PD	Total effective rate
Control group	40	14	8	10	8	22 (55.00%)
Research group	40	20	12	6	2	32 (80.00%)
χ^2^						5.698
P						0.017

### Serum-related factors of two groups

**Figure 1:**
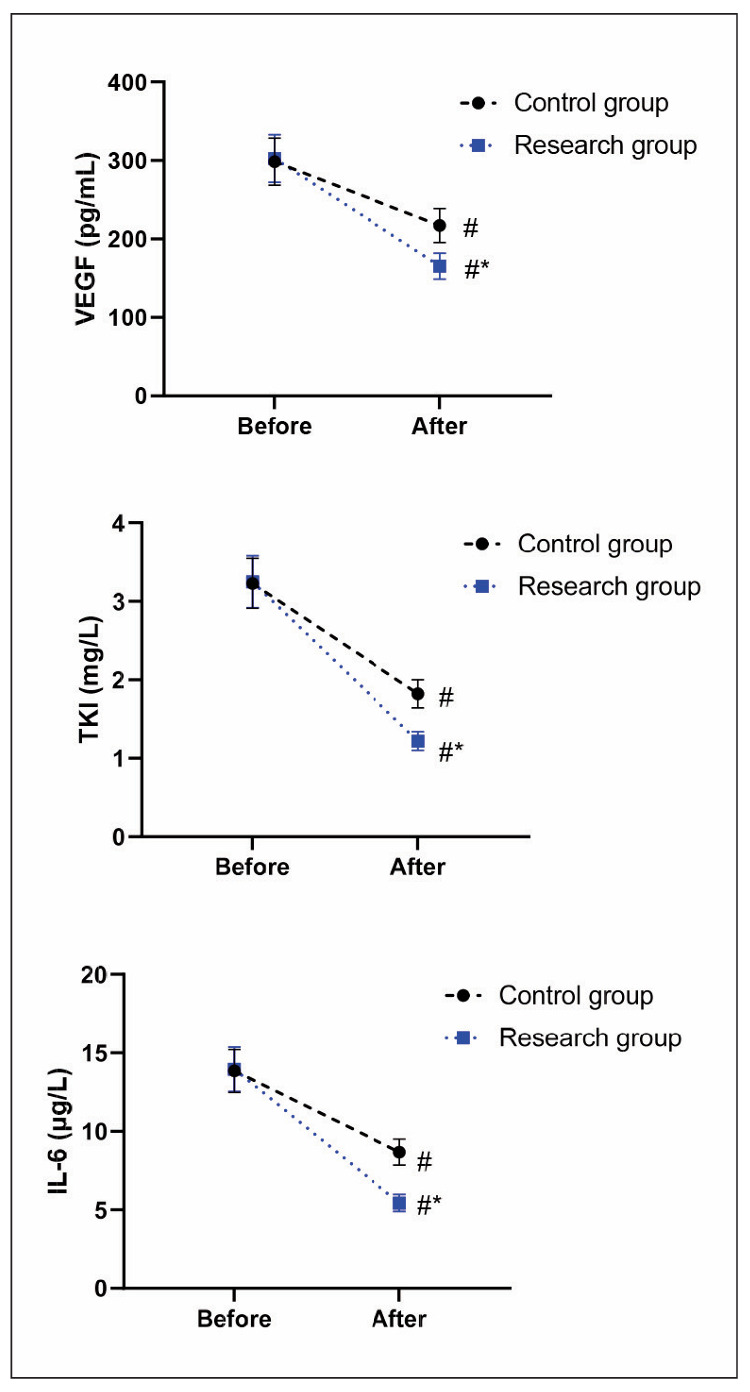
Diagnostic value of miR-124-3p, miR-944 and combined diagnosis.

Before therapy, no difference was seen in VEGF, TKI and IL-6 levels between the two groups (P>0.05). After therapy, VEGF, TKI as well as IL-6 levels declined in both groups (P<0.05), and those in the RG presented lower relative to the CG (P<0.05, Figure 1).

### Lymphocyte subsets index in both groups

**Figure 2:**
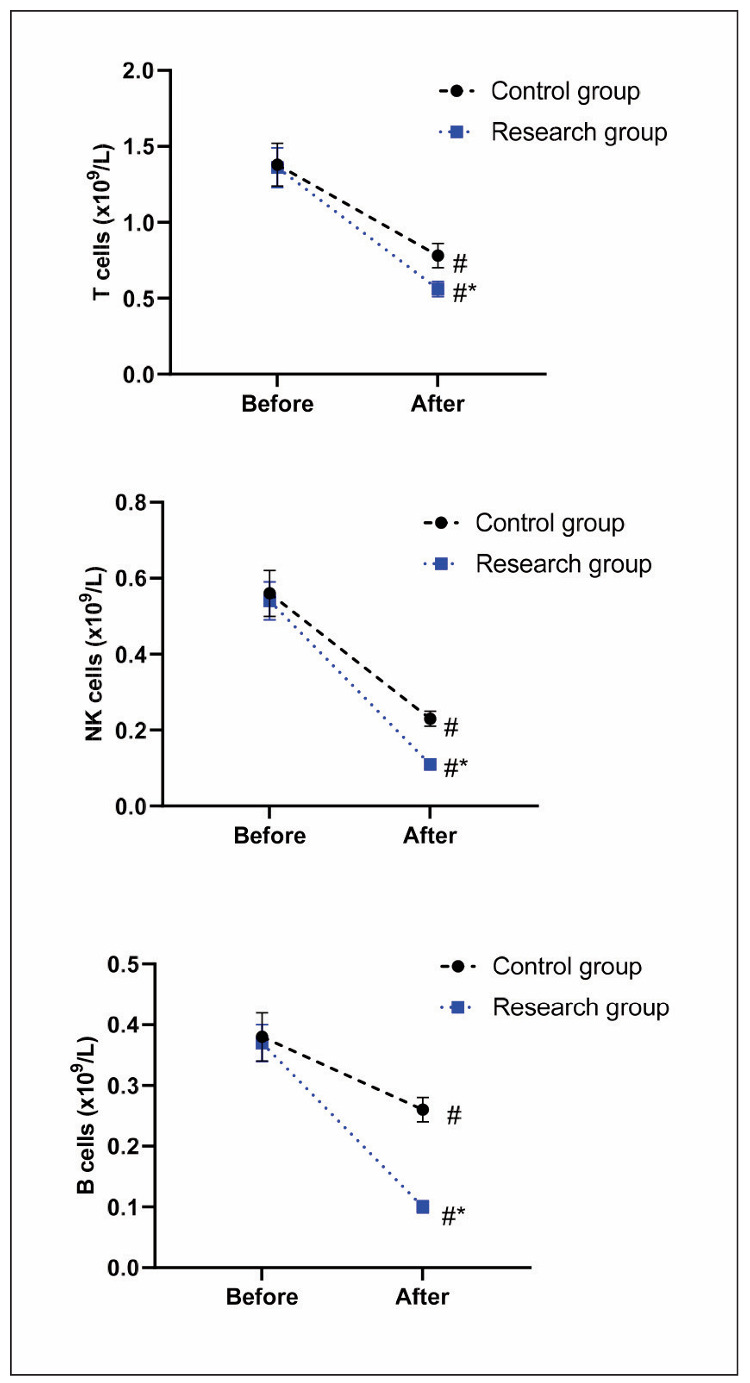
Lymphocyte subsets index in both groups. #P<0.05, in contrast to before therapy. *P<0.05, in contrast to CG.

Before therapy, no difference was seen in plasma T cells, NK cells, and B cells between the two groups (P>0.05). After therapy, plasma T cells, NK cells, and B cells declined in both groups (P<0.05), and those in the RG presented lower relative to the CG (P<0.05), as displayed in Figure 2.

### Occurrence of adverse reactions in both groups

Table 3 displayed that adverse reactions in the CG were 27.50% and that in the RG was 20.00%. There was no significant difference in the occurrence of adverse reactions between the two groups (P>0.05).

**Table 3:** Occurrence of adverse reactions in both groups.

Groups	Cases	Thrombocytopenia	Neutropenia	Mild<br>anaemia	Hepatotoxicity	Nausea and<br>vomiting	Neurotoxicity	Total<br>incidence rate
Control<br>group	40	2	1	2	1	3	2	11 (27.50%)
Research<br>group	40	1	1	2	1	2	1	8 (20.00%)
χ^2^								0.621
P								0.431

### Quality of life in both groups

**Figure 3:**
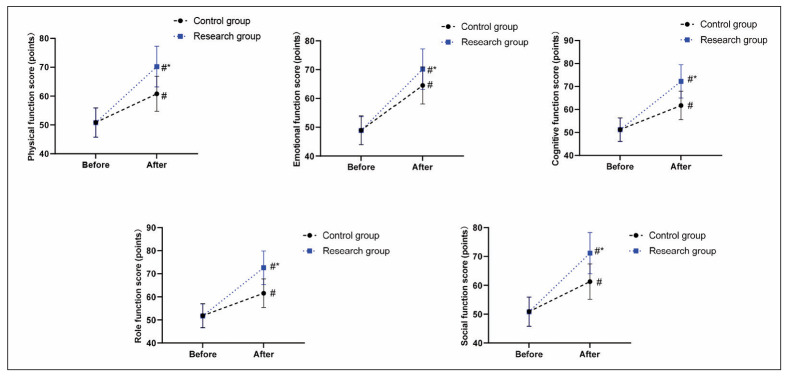
Quality of life in both groups. #P<0.05, in contrast to before therapy. *P<0.05, in contrast to CG.

Before therapy, no difference was seen in QLQ-C30 scores between the two groups (P>0.05). After therapy, QLQ-C30 scores were elevated in both groups (P<0.05), and those in the RG presented higher relative to the CG (P<0.05), as displayed in Figure 3.

## Discussion

DLBCL is a common clinical diffuse malignant proliferative disease of large B lymphocytes, typically characterised by rapid enlargement of painless masses [15]. Currently, chemotherapy is the primary treatment for this disease, and CHOP is the standard chemotherapy regimen for DLBCL, which has played a definite value in treating DLBCL [16]. However, long-term practice shows that the overall cure rate is still not ideal; some patients may appear drug-resistant, treatment ineffective or relapse phenomenon [17]. Therefore, it is essential to explore more effective treatments actively.

With the deepening of DLBCL research, some scholars have found that CD20 antigen is expressed in most tumour cells of DLBCL but not in hematopoietic stem cells in the body, and it does not bind to antibodies, so it has become a new therapeutic target of DLBCL [18]. Rituximab is a chimaera composed of a high-purity murine variable region (V region) and a human stable region (C region), wherein the murine variable region is extracted from the mouse anti-CD20 monoclonal antibody, namely IDE-C2B8 and the human stable region is human immunoglobulin IgG1 [19]. Rituximab can bind specifically to CD20 antigens to cause tumour cell lysis, inhibit tumour cell proliferation, lead to tumour cell apoptosis, and improve the sensitivity of tumour cells to chemotherapy drugs to improve drug resistance [20]. Its specific anti-tumour mechanism is as follows: (1) Antibody-dependent cellular cytotoxicity (ADCC): The Fc fragment of rituximab can bind to the Fc receptors of various effector cells (macrophages, T cells, NK cells, etc.) and activate effector cells to lose the cytotoxic perforin and granase, thus mediating antibody-dependent cytotoxicity [21]. (2) Complement-dependent cytotoxicity (CDC): In vitro studies have shown that rituximab can bind to C1q of the complement so that the complement protein can be fixed on the surface of antibody-coated tumour cells, mediating the complement-dependent cytotoxicity [22]. In vivo experiments have also confirmed this effect and found that rituximab binds to CD20, transforming it into a “fat raft” to promote the occurrence of complement reaction [23]. (3) Inducing B-cell apoptosis: Numerous experimental studies have confirmed that rituximab can induce tumour cell apoptosis. (4) Improving drug resistance of tumour cells: It has been confirmed that rituximab combined with CHOP chemotherapy can inhibit Bcl-2-related drug resistance in elderly DLBCL patients [24].

In our study, the results of Table 2 manifested that the total clinical effective rate of the RG presented better relative to the CG, suggesting that rituximab combined with chemotherapy could enhance the therapeutic effect. The reason is that chemotherapy is prone to drug resistance and other adverse phenomena, leading to unsatisfactory efficacy. Rituximab can promote the effective clearance of malignant B cells through complement-dependent cell-mediated cytotoxicity and apoptosis mechanisms, improve the sensitivity of lymphoma cells to chemotherapy drugs, facilitate cytotoxic drugs to kill tumour cells, and bind to transmembrane antigen CD20 to induce apoptosis of tumour cells [25], which was consistent with the previous study [26]. Consistently, Baek et al. suggested that adding rituximab to conventional chemotherapy for CD20-positive acute lymphoblastic leukaemia is effective and tolerable [27].

The pathogenesis of DLBCL is related to immune disorders [28]. Plasma T cells, NK cells, and B cells are indicators of immune function and can reflect the body’s immune function [29]. Elevated levels of these cells suggest that patients’ immune function is seriously impaired [30]. In addition, VEGF is a key factor in angiogenesis, and its elevated serum level indicates an increase in the rate of neovascularisation, which is conducive to tumour recurrence or metastasis [31]. TK1 is a special cytoplasmic kinase catalyses thymidine to 1-phosphothymidylate and is an essential precursor for DNA synthesis in tumour cells [32]. When tumour cells proliferate, the normal cell cycle regulation is broken, and TK1 is released into the blood so that the TK1 can reflect the proliferation rate of tumour cells [33]. IL-6 belongs to a common inflammatory factor, which can reflect the body’s inflammatory response and its level can stimulate disease progression [34]. In our study, the results of Figure 1 and Figure 2 displayed that after therapy, levels of VEGF, TKI and IL-6 levels, as well as plasma T cells, NK cells together with B cells in the RG presented lower relative to the CG, indicating that rituximab combined with chemotherapy could better reduce the inflammatory response and promote the immune function of DLBCL patients. Similarly, rituximab has been reported to relieve inflammatory response and enhance the immune function in treating DLBCL [35] [36].

In addition, the results of Table 3 indicated no statistical significance in the occurrence of adverse reactions between the two groups, implying that the addition of rituximab did not significantly elevate the risk of adverse reactions and had a good safety profile [37]. Likewise, Song et al. indicated that chemotherapy regimens containing rituximab may be a safe option for use as the treatment of DLBCL patients [38]. In our study, the results of Figure 3 indicated that after therapy, QLQ-C30 scores were elevated in both groups, and those in the RG presented higher relative to the CG, implying that rituximab in combination with chemotherapy could better promote the quality of life of DLBCL patients. The reason is that rituximab has a more effective anti-tumour effect, reduces the impact of DLBCL symptoms on patients’ lives, and ultimately improves the quality of life, similar to research proposed by Lu et al. [39].

## Conclusion

Rituximab combined with chemotherapy is effective in treating DLBCL patients, which can reduce serum-related factors promoting immune function and quality of life. Our study may provide compelling evidence for supporting the therapeutic regimen of rituximab combined with chemotherapy in DLBCL patients.

## Dodatak

### Funding

None.

### Ethical consideration

The Ethics Committee of our institution approved the study, and informed consent was obtained from all patients.

### Author contribution

Lihua Tian and Baoan Luo conceived and designed the study and collected and analysed the data. Jun Tang and Jiagui Ye prepared the manuscript. All authors mentioned in the article approved the manuscript.

### Conflict of interest statement

All the authors declare that they have no conflict of interest in this work.

## Dodatak

### List of abbreviations

Complete response, CR;<br>Control group, CG;<br>Cyclophosphamide, doxorubicin, vincristine, and prednisone, CHOP;<br>Diffuse large B-cell lymphoma, DLBCL;<br>Interleukin-6, IL-6;<br>Non-Hodgkin’s lymphoma, NHL;<br>Natural killer, NK;<br>Partial response, PR;<br>Quality of Life Core Questionnaire-Core 30, QLQ-C30;<br>Research group, RG;<br>Stable disease, SD;<br>Thymidine kinase 1, TK1;<br>Vascular endothelial growth factor, VEGF
